# Neuroprotection of NAD^+^ and NBP against ischemia/reperfusion brain injury is associated with restoration of sirtuin-regulated metabolic homeostasis

**DOI:** 10.3389/fphar.2023.1096533

**Published:** 2023-03-28

**Authors:** Xin-Xin Wang, Guang-Hui Mao, Qi-Qi Li, Jie Tang, Hua Zhang, Kang-Lin Wang, Lei Wang, Hong Ni, Rui Sheng, Zheng-Hong Qin

**Affiliations:** ^1^ Department of Pharmacology and Laboratory of Aging and Nervous Diseases, Jiangsu Key Laboratory of Neuropsychiatric Diseases, College of Pharmaceutical Sciences of Soochow University, Suzhou, China; ^2^ Institute of Pediatric Research, Children’s Hospital of Soochow University, Suzhou, China; ^3^ Hefei Knature Bio-pharm Co., Ltd., Hefei, China

**Keywords:** NAD^+^, NBP, cerebral ischemia-reperfusion, mitochondria, SIRT3, SIRT1, acetylation

## Abstract

**Background:** Ischemic stroke seriously threatens human health because of high rates of morbidity, mortality and disability. This study compared the effects of nicotinamide adenine dinucleotide (NAD^+^) and butylphthalide (NBP) on *in vitro* and *in vivo* ischemic stroke models.

**Methods:** Transient middle cerebral artery occlusion/reperfusion (t-MCAO/R) model was established in mice, and the cultured primary cortical neurons were subjected to oxygen-glucose deprivation/reoxygenation (OGD/R). Cerebral infarct volume, neurobehavioral indices, antioxidant activity, ATP level and lactic acid content were determined. The neuroprotective effects of NAD^+^ or NBP were compared using sirtuin inhibitor niacinamide (NAM).

**Results:** Intraperitoneal injection of NBP within 4 h or intravenous injection of NAD^+^ within 1 h after t-MCAO/R significantly reduced the volume of infarcts, cerebral edema, and neurological deficits. Administration of NAD^+^ and NBP immediately after t-MCAO/R in mice showed similar neuroprotection against acute and long-term ischemic injury. Both NAD^+^ and NBP significantly inhibited the accumulation of MDA and H_2_O_2_ and reduced oxidative stress. NAD^+^ was superior to NBP in inhibiting lipid oxidation and DNA damage. Furthermore, although both NAD^+^ and NBP improved the morphology of mitochondrial damage induced by ischemia/reperfusion, NAD^+^ more effectively reversed the decrease of ATP and increase of lactic acid after ischemia/reperfusion compared with NBP. NAD^+^ but not NBP treatment significantly upregulated SIRT3 in the brain, but the sirtuin inhibitor NAM could abolish the protective effect of NAD^+^ and NBP by inhibiting SIRT1 or SIRT3.

**Conclusions:** These results confirmed the protective effects of NAD^+^ and NBP on cerebral ischemic injury. NBP and NAD^+^ showed similar antioxidant effects, while NAD^+^ had better ability in restoring energy metabolism, possibly through upregulating the activity of SIRT1 and SIRT3. The protection provided by NBP against cerebral ischemia/reperfusion may be achieved through SIRT1.

## 1 Introduction

Stroke is brain damage result from a sudden rupture of brain blood vessel or by a blockage of the blood vessel that prevents blood from flowing to the brain. Approximately 15 million people in the world suffer from stroke each year (Toman et al., 2019), among them are over 5 million people die every year, while another 5 million suffer from long-term disability (Wu et al., 2019). Therefore, stroke seriously threatens human health because of the high rates of morbidity, mortality, and disability (Kumaret al., 2010; Regenhardt et al., 2018). About 80% of strokes are due to cerebral artery occlusions (Campbell et al., 2019).

The brain relies on blood flow to continuously deliver oxygen and glucose. In the event of an ischemic stroke, the interruption of blood supply to the brain leads to hypoglycemia and hypoxia in the brain, and the energy depletion of neurons. The initiation of anaerobic respiration in cells causes lactic acid accumulation, abnormal release of neurotransmitter, and excessive production of reactive oxygen species, leading to acute brain injury (Kalogeris et al., 2016). If the blood flow in the brain is resumed in a short period, that is, the supply of oxygen and glucose is restored, the cerebral ischemic damage can be alleviated. However, if the reperfusion of cerebral blood flow exceeds the time limit of reperfusion, the brain injury will be exacerbated. This is due to the uncoupling of mitochondrial oxidative phosphorylation after reperfusion, which produces a large number of free radicals. The free radicals may react with lipids, proteins, nucleic acids, and other macromolecules, causing a series of peroxidative damage. Compared to glia and vascular cells, neurons are more susceptible to damage (Iadecola and Anrather, 2011), and finally various programs of cell death are activated, including necrosis, pyrolysis, apoptosis, ferroptosis or autophagy, etc. (Eltzschig and Eckle, 2011; Sekerdag et al., 2018).

At the present, stroke patients still benefit from thrombolytic therapy early in the course of their illness with tissue plasminogen activator (t-PA) (Campbell et al., 2019). However, the clinical use of t-PA is very limited for two reasons. Firstly, the treatment time window of t-PA is narrow, only effective within 3 h after ischemia. Secondly, there is a high risk of intracranial hemorrhage (Dewar and Shamy, 2020). In addition, acute brain injury and complications caused by stroke, including heart failure (Chen et al., 2017) and cognitive impairment after stroke (Cuartero et al., 2019), have increased the difficulty of stroke treatment. As a result, in clinical practice, a new and effective drug is therefore urgently needed.

The mitochondria, cytoplasm, and nucleus contain abundant with nicotinamide adenine dinucleotide (NAD^+^), sometimes referred to as coenzyme I. NAD^+^ is necessary cofactor for many metabolic pathways, including glycolysis, β-oxidation of fatty acids, tricarboxylic acid cycle and oxidative phosphorylation (Imai and Guarente, 2014). In mammalian cells, NAD^+^ is consumed mainly in three ways (Rajman et al., 2018; Migaud and Lee, 2019): 1) NAD^+^ activates sirtuins to improve metabolic efficiency and enhances antioxidant capacity (Imai and Guarente, 2014). 2) NAD^+^ activates PARPs pathway for DNA repair. 3) NAD^+^ activates CD38 pathway to mediate calcium signaling. In the process of aging, the decline of NAD^+^ level can cause defects in nuclear and mitochondrial function, associated with a variety of age-related illnesses (Cimaglia et al., 2020), such as Alzheimer’s disease (AD) (Hou et al., 2018), Parkinson’s disease (PD) (Jia et al., 2008), Huntington’s disease (HD) (Hathorn et al., 2011) and amyotrophic lateral sclerosis (ALS) (Harlan et al., 2016), *etc.* Restoring NAD^+^ level by supplementing NAD^+^ or its intermediates can significantly protect these age-related diseases (Kulikova et al., 2018). It has been previously reported that NMN, a NAD^+^ precursor, can inhibit mitochondrial fission and ROS generation in mice with cerebral ischemia, thereby playing a neuroprotective effect against cerebral ischemia (Klimova et al., 2020). Our previous studies and other investigators have shown that exogenous NAD^+^ treatment in the early stage of cerebral I/R in mice can alleviate neuronal injury by inhibiting oxidative stress (Li et al., 2011; Huang et al., 2018).

DL-3N-Butylphthalide (NBP) is an oil-like liquid drug independently researched by the Chinese Academy of Medical Sciences, Institute of Materia Medica for the treatment of ischemic stroke (Dong and Feng, 2002) (Shan Huang et al., 2021). Animal studies have shown that NBP can obstruct multiple pathological processes of brain injury caused by ischemic stroke (Chen X. Q. et al., 2019). NBP boosts microcirculation in the ischemic brain by increasing capillaries (Ye et al., 2019). NBP alleviates brain edema (Zeng et al., 2020), reduces the infarct area and inhibits neuronal apoptosis and neuroinflammation induced by focal cerebral ischemia in rodents (Yang et al., 2019). NBP also inhibit cerebral thrombosis and platelet aggregation. Mechanically, NBP may increase the levels of NO and PGI_2_ by reducing the content of arachidonic acid in cerebrovascular endothelium. NBP inhibits the release of glutamate and reduces intracellular calcium (Wang et al., 2018). NBP inhibits oxygen free radicals, enhances antioxidant enzyme activity, and improves energy depletion after cerebral ischemia (Yan et al., 2017; Zhao et al., 2019). Beyond that, NBP inhibits apoptosis by inhibiting caspase activation and reducing endogenous cytochrome C and apoptosis-inducing factors (Min et al., 2014) (Chen J. et al., 2019). NBP has also been shown to improve mitochondrial function and inhibit autophagy (Zhao et al., 2016). The results of clinical studies showed that NBP is effective in improving central nervous system function after injury and promote the recovery of impaired the neurological function of patients with acute cerebral ischemia. NBP thus is widely used in the domestic and international markets due to its neuroprotective effect on ischemic cerebrovascular diseases.

The purpose of this study was to investigate the effects of NAD^+^ and NBP on ischemic stroke in mice with transient middle cerebral artery occlusion/reperfusion (t-MCAO/R) and cultured primary cortical neurons subjected to oxygen glucose deprivation/reoxygenation (OGD/R). The difference of the effects of NAD^+^ and NBP on energy metabolism and oxidative stress was further studied. The results showed that NAD^+^ and NBP had similar neuroprotective and antioxidant effects in cerebral ischemia, while NAD^+^ had better ability in restoring energy metabolism, possibly through upregulating the activity of SIRT1 and SIRT3. The protection provided by NBP against cerebral ischemia/reperfusion may be achieved through SIRT1.

## 2 Materials and methods

### 2.1 Animals

JOINN Laboratories Inc. (Suzhou, China) provided the ICR mice (male, 25–28 g) and the ICR pregnant mice (female, 17–18 days of gestation). All animal procedures were approved by Soochow University’s Ethical Committee and followed the guidelines from the National Institutes of Health for the Use and Care of Laboratory Animals. The animals were kept under controlled conditions of humidity and temperature with a 12-h lighting cycle and given a standard diet and water *ad libitum*.

### 2.2 Surgical procedure of transient middle cerebral artery occlusion in mice

The model of T-MCAO/R was established in mice according to the methods described before (Tullis, 1997). In brief, groups of mice were selected at random. A 1.5% isoflurane-inhaled anesthetic was used to anesthetize the mice. We exposed and separated the right common carotid artery as well as the internal and external carotid arteries of mice. The distal end of the external carotid artery was incised and a silicone-coated nylon monofilament (Doccol Corporation 602356PK5Re) was inserted into the internal carotid until it reached the beginning of the middle cerebral artery (MCA) to cut off its blood supply. Using a doppler flowmeter, we monitored the blood flow for 2 h before removing the suture and restoring blood flow. Mice in the sham group were treated as the same as those in the ischemia group, except those inserted suture. A heating pad was used to control the body temperature of the mice at 37°C during the surgery.

### 2.3 *In vivo* and *in vitro* drug administration

NAD^+^ was purchased from Sigma-Aldrich (United States, N7004). NBP was purchased from National Center for Standard Materials (https://www.bwsm.org/, No. 101035). NAM was purchased from Selleck (S1899). For *in vivo* studies, NAD^+^ (50 mg/kg) was dissolved in 0.9% saline and injected through tail vein at different time after reperfusion. Different concentrations of NBP injection were compounded according to the NBP dissolution formula: 10% DMSO +40% PEG300 + 5% tween-80 + 45% saline. Mice were administrated by intraperitoneal injection of NBP at different time after reperfusion. Mice in control group were administrated with the same volume of vehicle. NAM (100 mg/kg) was dissolved in 0.9% saline and injected intraperitoneally at different time after reperfusion. For *in vitro* studies, NAD^+^ was dissolved in distilled water and prepared into 1.5 M stock solution, diluted to 15 mM before use. NBP was prepared into 5 mM stock solution with DMSO and dilute to 5 μM before use. NAM was dissolved in PBS and prepared into 100 mM stock solution, diluted to 10 mM before use. DMSO concentration was less than 0.1%.

Cell culture, oxygen-glucose deprivation and reperfusion, cell viability and cell toxicity determination.

The primary cortical neurons were isolated from ICR fetal mice (E17-18) according to methods described earlier (Marutani et al., 2012). Neurobasal medium (21103-049, Gibco) containing 2% B27 (17504-044, Gibco), 0.25% glutamine (200 mM, G0374, Sango, Shanghai) and 0.25% glutamate (10 mM, G8415, Sigma) were used to culture the primary cortical neurons. For oxygen-glucose deprivation and reperfusion (OGD/R) model, the culture medium was replaced with sugar-free Hank’s balanced salt solution (HBSS: 116 mM Tris, 0.8 mM MgSO4, 5.4 mM KCl, 1 mM KH2PO4, 1.8 mM CaCl2, 26.2 mM NaHCO3, 2.38 g/L Hepes, pH7.4), and the neurons were placed in Modular Incubator Chamber (MC-101, Billups-Rothenberg) for 4 h at 37°C, then the neurons were restored with the original culture medium under normaxia. For cell viability determination, after reoxygenation for 24 h, CCK-8 (FC101-04, TRANS) reagent was added to the culture and incubated at 37°C for 2 h. The absorbance was detected at 450 nm wavelength. For cell toxicity determination, after reoxygenation for 24 h, LDH (C0016, Beyotime) reagent was added to the culture and incubated at 25°C for 30 min. The absorbance was detected at 490 nm wavelength.

### 2.4 Measurement of infarct volume

After t-MCAO/R for 24 h, the mice were sacrificed. Brain tissue was removed, and we froze it at −30°C for about 15 min. The olfactory bulb, cerebellum and lower brainstem of mice were removed, and then the coronal uniform cutting was performed, and the brain was divided into 5 pieces (2 mm/piece). Then the brain slices were placed in an ampoule containing 1% 2,3, 5-triphenyltetrazolium chloride (TTC) dye and incubated in the dark at 42°C for 10 min. Infarct volume is expressed as the ratio of infarct area in white to hemibrain area.

### 2.5 Assessment of neurological score and behavioral tests

After 24 h of reperfusion, neurological scores were evaluated by an investigator who was not aware of the experimental conditions in each group. We evaluated neurological deficits using the previously explained 5-point scale (Bederson et al., 1986): 0, no symptoms of neurological impairment; 1, inability to fully extend the contralateral forelimb; 2, when walking, the opposite side rotates, and the phenomenon of “rear-end collision” appears; 3, unsteady standing, toppling to the opposite side; 4, Unable to walk spontaneously, impaired consciousness. At 28 days after cerebral ischemic insult, the balance beam, rotary rod, and new object recognition test were used to detect the neurological recovery of the mice.

A balance beam was used to assess the balance coordination ability of the mice. The apparatus used was a wood product with a height of 50 cm, a length of 75 cm, and a width of 10 cm. One end was randomly selected as the starting point for the mice and the other end as the end point for the feed. First, the mice were placed on one end of the apparatus for 3 min to acclimatize, and the mice were guided to the other end with the feed. When the mice could crawl to the other end independently, they were considered to have learned, and then sprayed with alcohol. The test was performed 4 h later. The mice were placed at the starting position of the training period with no more feed at the other end, and the time the mice passed the balance beam was recorded with a stopwatch, and the two tests were averaged.

Rotary rod is commonly used to assess motor coordination and balance. First, the mouse was placed on the rotarod to explore freely for 3-min, and the rotation speed of the rotarod was started at 12 rpm/min. The mouse tail was supported by the hand to help it walk on the rotarod. Mice were considered to have learned when they could walk continuously for 1 min. The test was performed 4 h later. The time the mice held on the rotorod during a 5-min period of uniform acceleration from 12 rpm/min to 40 rpm/min was recorded, and the two tests were averaged.

New object recognition was used to evaluate the non-spatial memory ability of mice. First, two cubes were placed diagonally inside the box and at an appropriate passing distance from the box wall, and the mice were allowed to move freely inside the box for 5-min. The test was performed 2 h later. One of the cubes was replaced with a triangle, and the exploration activity of the mice was recorded for 5 min. After the mice had completed exploration, alcohol was sprayed to remove the odor. At the end of the experiment, the exploratory behavior of the mice towards the two objects was recorded by playback of the video. Index = time spent exploring new objects/total time spent exploring old and new objects.

### 2.6 Measurement of brain water content

The olfactory bulb, cerebellum and lower brainstem were removed after I/R 24 h. The wet weight of the brain was measured, and the dry weight was measured after being roasted at 37°C for 72 h. The percentage of brain water content = (wet weight-dry weight)/wet weight ×100%

### 2.7 Morphological observation of mitochondria

At 6 h after cerebral reperfusion, the mice were sacrificed and subjected to cardiac perfusion with PBS and 4% paraformaldehyde. Then the right penumbra with a volume of 1 mm^3^ was obtained and fixed in electron microscopy fixative solution (G1102, Servicebio). The tissue was dehydrated by gradient alcohol and acetone, then permeated and embedded with EMBed 812 (90529-77-4, SPI), and polymerized for 48 h. The tissue was cut into thin slices with a thickness of 60–80 nm by Ultra microtome (Leica UC7, Leica), stained and observed with transmission electron microscopy (HT7800, HITACHI). The area of mitochondria was calculated using ImageJ.

### 2.8 Determination of MDA, ATP, lactic acid and H_2_O_2_ in brain and cortical neurons

For *in vivo* studies, the mice were sacrificed at 3 h after cerebral ischemia/reperfusion. The brains were removed to obtain the intact ischemic lateral cortical tissue, and the tissue was homogenated on ice with a homogenizer. After centrifugation at 4°C, the supernatant was taken and the levels of MDA, lactic acid (LD), ATP and H_2_O_2_ were detected with MDA assay kit (S0131, Beyotime), LD assay kit (A019-2-1, NJJCBIO), ATP assay kit (S0026, Beyotime) and H_2_O_2_ assay kit (S0038, Beyotime) in accordance with the manufacturer’s recommendations. For *in vitro* studies, the ATP levels of primary cortical neurons were detected with ATP Assay kit 3 h after OGD/R. The operating principle of ATP Assay Kit is based on the fact that firefly luciferase (also called luciferase) catalyzes luciferin to produce fluorescence and requires ATP to provide energy. When both firefly luciferase and luciferin are in excess, the production of fluorescence is proportional to the concentration of ATP within a certain concentration range. This allows highly sensitive detection of ATP concentration. The operating principle of hydrogen peroxide Assay Kit is that hydrogen peroxide can oxidize divalent iron ions to trivalent iron ions, and then form a purple product with xylenol orange in a specific solution.

### 2.9 ROS/mitoROS were observed under fluorescence microscopy

For ROS assay, the cells were incubated with fluorescent probe DCFH-DA (S0033S, Beyotime, 1:1,000) at 37°C for 30 min. For mitoROS assay, the cells were incubated with the red fluorescent probe mitoSOX and the MitoBright LT Green fluorescent probe (40778ES50, Yeasen, 1:1,000) at 37°C for 30 min. Then the cells were counterstained with Hoechst33342 for 10 min. The immunofluorescence was then observed with a Nikon confocal microscope (AIR HD25, ABI).

### 2.10 Evaluate the degree of acetylation

In mice, MCAO was performed for 2 h. NAD^+^ and NBP were administered immediately after reperfusion. To evaluate acetylation of NDUFA9 in cortex 3 h after cerebral ischemia/reperfusion in mice. Cortex were dissected and lysed in IP buffer [150 mM NaCl, 50 mM Tris-HCl (pH 7.4), 1 mM EDTA, 1% Triton X-1000, 0.1% SDS, and EDTA-free complete protease inhibitor (Roche)]. The lysates were precleaned with protein A/G-agarose (Biomake B23201) for 1–3 h, incubated with anti-NDUFA9 (#ab128744, Abcam, Cambridge, United Kingdom) overnight. After washing away unbound antibody, the lysates were incubated with protein G-agarose for 4–8 h. The immunoprecipitates were analyzed by Western blotting using antibody against Kac (#9441, Cell Signaling Technology, Danvers, United States).

### 2.11 Western blot analysis

The Western blotting was conducted as described earlier (Huang et al., 2018). Briefly, the ischemic cortex was collected at different time after reperfusion, homogenized in tissue lysing buffer. Proteins were separated with 8%–12% SDS-polyacrylamide gel and transferred to nitrification cellulose membranes with glycine transfer buffer. The membrane was sealed with 5% skim milk and protein was detected with primary antibody, including mouse anti-nitrotyrosine (#05233, Millipore, Darmstadt, Germany), mouse anti-γH2. AX (#ab2893, Abcam, Cambridge, United Kingdom), rabbit anti-4-hydroxynonenal (#ab46545, Abcam, Cambridge, United Kingdom), anti-SIRT3 (#2627, Cell Signaling Technology, Danvers, United States), anti-PGC-1α antibody (#SC13067, Santa Cruz, Dallas, United States), and mouse anti-GAPDH (#ab9484, Millipore, Darmstadt, Germany), anti-β-Actin (#A5441, Sigma, Darmstadt, Germany). After incubating overnight at 4°C, the membrane was incubated with secondary antibodies including anti-mouse IgG or anti-rabbit IgG (Li-Cor Bioscience, Lincoln, NE, United States). The protein bands were visualized using the Odyssey Infrared Imaging System (Li-Cor Bioscience). After the images were collected, the protein expression was finally determined using ImageJ Launcher software (National Institutes of Health, Bethesda, MD, United States) and normalized to a loading control GAPDH.

### 2.12 Statistical analysis

The results were statistically analyzed with GraphPad Prism 8 software. All data were expressed as mean ± standard deviation (SD). Multi-mean comparison was performed using One-way ANOVA’s followed by Tukey’s test, and two-mean comparison was performed using *t*-test. *p* < 0.05 was considered as statistically significant.

## 3 Results

### 3.1 Effect of NBP administration on cerebral ischemic injury in mice

**FIGURE 1:**
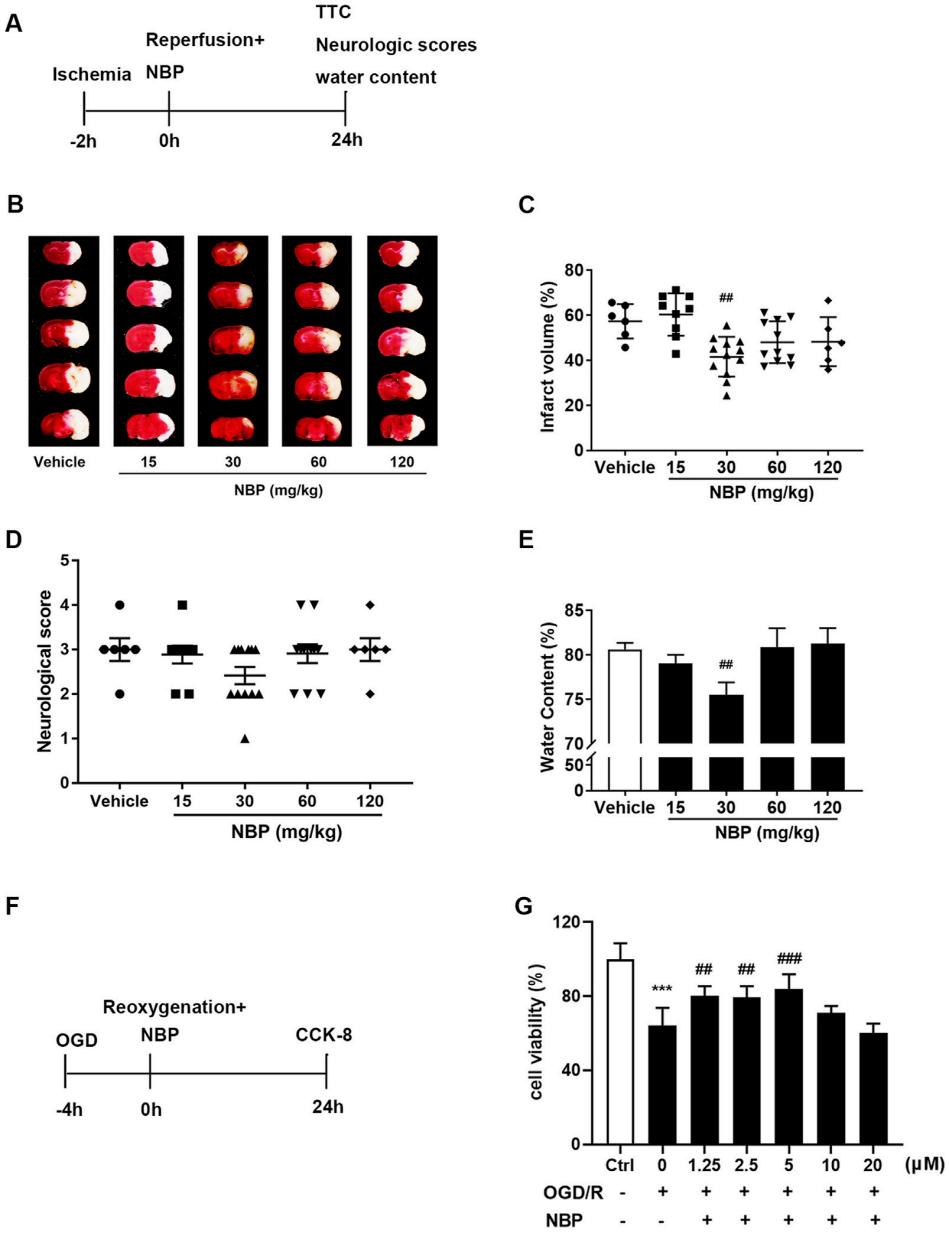
Effects different doses of NBP on ischemic stroke. **(A)** In mice, MCAO was performed for 2 h, and NBP (15, 30, 60 and 120 mg/kg) was injected intraperitoneally as soon as possible after reperfusion. Representative images of 2,3,5-triphenyltetrazolium chloride (TTC) staining **(B)** and quantitative analysis of infarct volume **(C)** 24 h after reperfusion. The neurological deficits **(D)** and water content **(E)** 24 h after reperfusion. **(F)** Glucose and hypoxia were taken away from the primary cortical neurons for 4 h, and different concentration of NBP were administered transiently after reoxygenation. After 24 h of reperfusion, the survival rate of neurons was detected with CCK-8 kit. **(G)** Cell viability (%) in each group. n = 6 per group. ^*##*^ *p* < 0.01, ^*##*^ *p <* 0.01 and ^*###*^ *p <* 0.001 vs. vehicle or OGD/R, ***p < 0.001 vs. ctrl by One-way ANOVA followed by Tukey’s multiple comparisons test. Data are expressed as mean ± SD.

To confirm the neuroprotective effect of NBP on ischemic brain injury in mice, we referred to the references (Sun et al., 2017; Qin et al., 2019) to select the dosage of intraperitoneal injection of NBP (15, 30, 60, 120 mg/kg). TTC staining, neurological scores and brain water content were used to detect the effects of different doses of NBP on the cerebral injury in mice with ischemic stroke. Based on the results, it was found that compared to vehicles, the intraperitoneal injection of 30 mg/kg NBP immediately after t-MCAO/R significantly reduced the cerebral infarct volume (Figures 1B, C) and alleviated cerebral edema in mice (Figure 1E), but NBP did not show effect on the neurological scores (Figure 1D). NBP 60 and 120 mg/kg also showed a tendency to decrease infarct volume but did not reach significance, which may be due to the severe injury in the t-MCAO/R model in this study. The primary cortical neurons were treated with OGD/R, and it was found that 5 μM NBP can significantly improve the cell viability of cultured neurons (Figures 1F, G).

**FIGURE 2:**
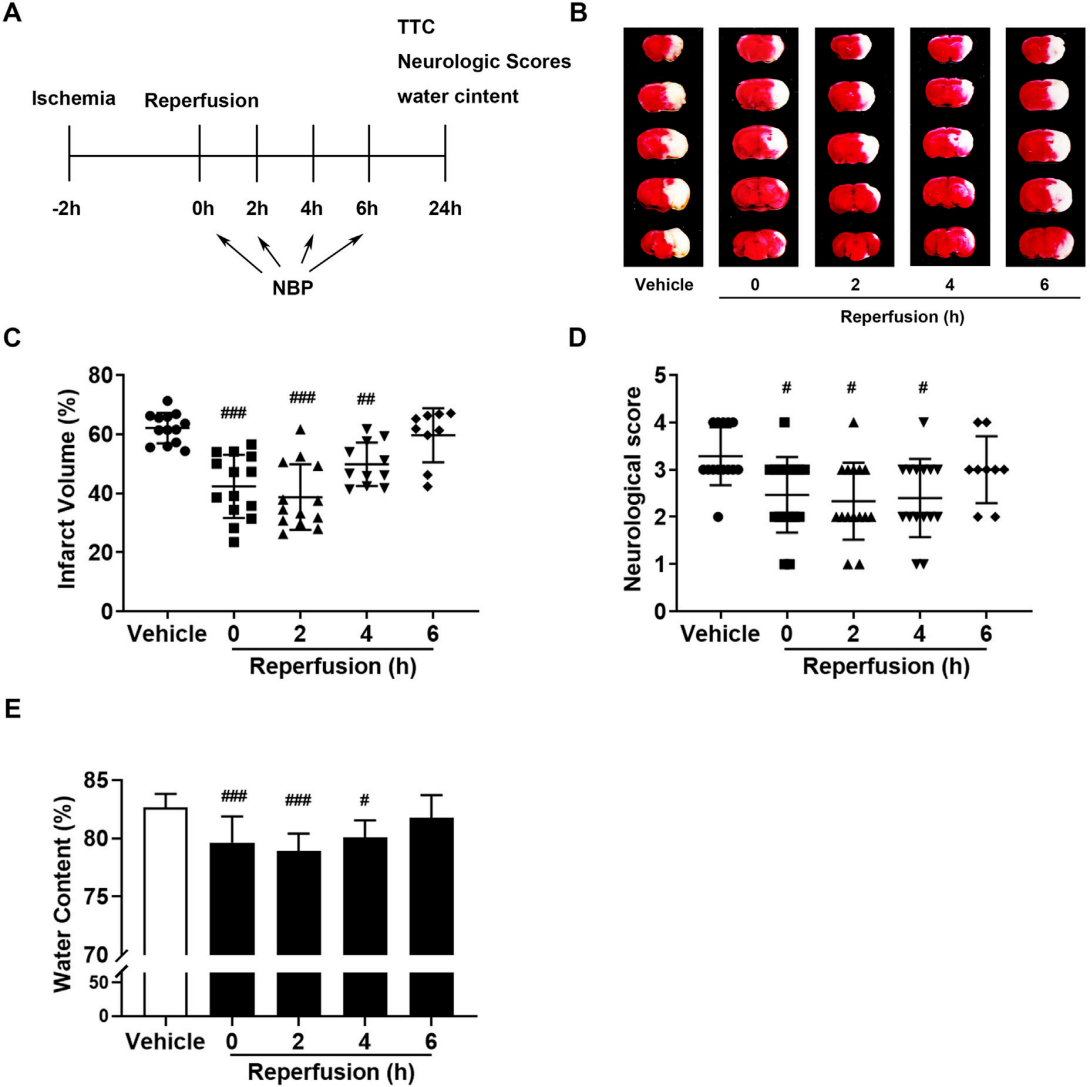
The therapeutic time window of NBP in the treatment of ischemia-reperfusion injury in mice. **(A)** In mice, MCAO was performed for 2 h, and NBP (30 mg/kg) was managed by intraperitoneal injection at 0, 2, 4 and 6 h after reperfusion. **(B, C)** Representative images of brain infarction volume detected with TTC staining. The neurological deficit score **(D)** and brain water content **(E)** 24 h after reperfusion. *p < 0.05, **p < 0.01 and ***p < 0.001 vs. vehicle, by One-way analysis of ANOVA followed by Tukey’s multiple comparisons test. Data were expressed as the mean ± SD. There were at least nine animals in each group.

To study the therapeutic window of NBP for ischemic stroke, we intraperitoneally injected NBP (30 mg/kg) into mice after reperfusion at different times (Figure 2A). Based on the TTC staining results, we can find that compared to the vehicles, NBP significantly reduced the cerebral infarct volume at 0 h, 2 h, and 4 h after reperfusion (Figures 2B, C). The cerebral water content of t-MCAO/R was significantly reduced by NBP within 4 h after reperfusion for 24 h (Figure 2E). Similar results were obtained from neurobehavioral symptoms (Figure 2D). From this part of the results, we can conclude that NBP had a relatively long treatment time window in the treatment of ischemic stroke, and it was useful within 4 h after reperfusion.

### 3.2 Comparison of the effects of NBP and NAD^+^ on cerebral ischemic injury

**FIGURE 3:**
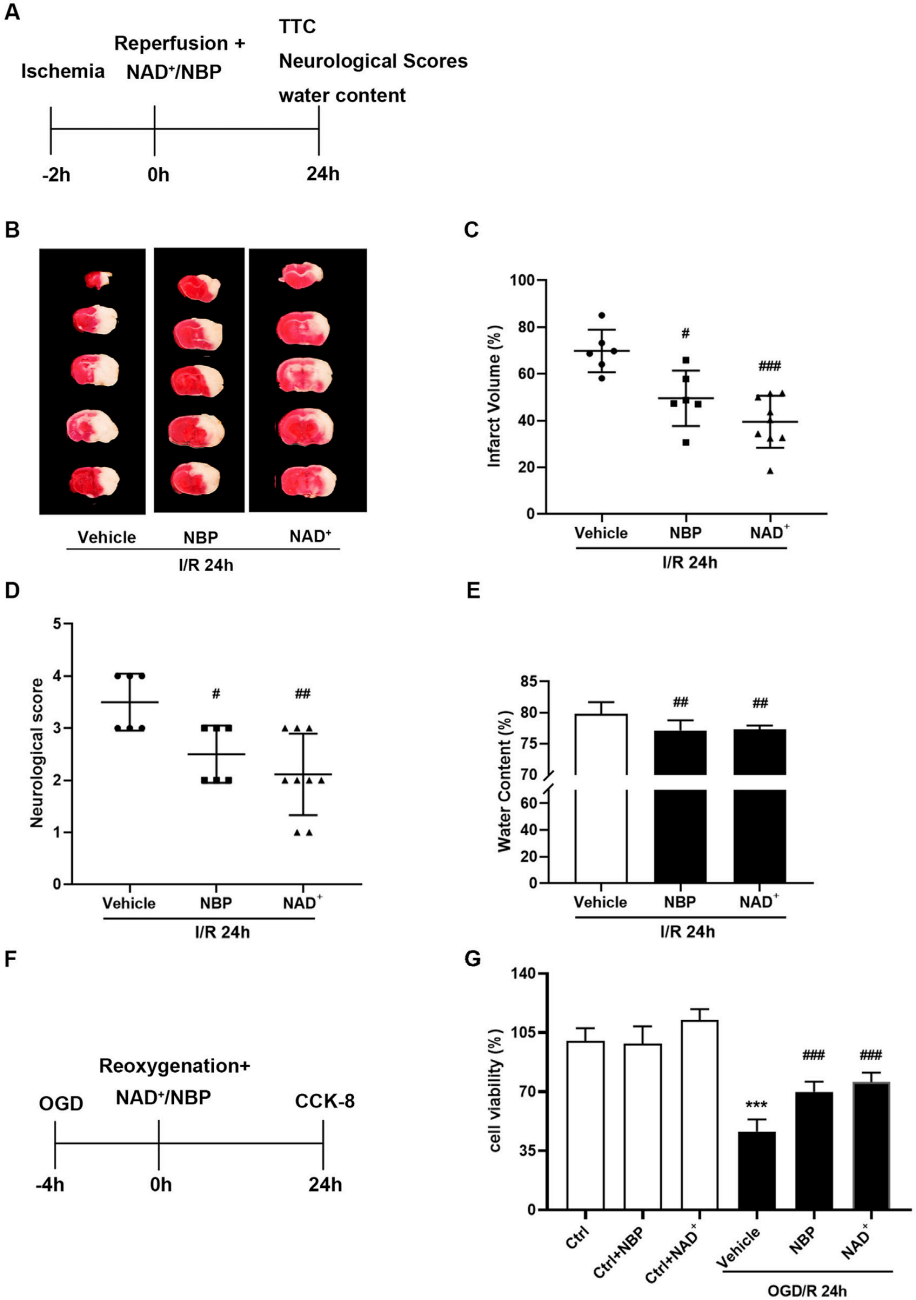
Comparison of the effects of NBP and NAD^+^ in the treatment of ischemic stroke. **(A)** In mice, MCAO was performed for 2 h, and NBP (30 mg/kg) or NAD^+^ (50 mg/kg) were administrated to mice as soon as possible after reperfusion. MCAO/R-treated mice were sacrificed 24 h after the procedure. **(B, C)** TTC staining was used to measure the cerebral infarct volume. **(D)** The neurological deficit score at 24 h after reperfusion. **(E)** The brain water content at 24 h after reperfusion. There were at least six animals in each group. **(F)** Glucose and hypoxia were taken away from the primary cortical neurons for 4 h, and 15 mM NAD^+^ or 5 μM NBP were administered transiently after reperfusion. After 24 h of reperfusion, the viability of the cells was determined using the CCK-8 kit. **(G)** Cell viability in each group. n = 6 per group. ***p < 0.001 vs. ctrl, ^*#*^ *p <* 0.05, ^*##*^ *p <* 0.01, ^*###*^ *p <* 0.001 vs. vehicle by One-way analysis of ANOVA followed by Tukey’s multiple comparisons test. Data were expressed as the mean ± SD.

Our previous results showed that the therapeutic dose of NAD^+^ for cerebral I/R injury in mice is 50 mg/kg, and the therapeutic effect can only be achieved within 1 h after reperfusion. Meanwhile, NAD^+^, with the concentration of 15 mM, showed a significant protective effect against OGD/R injury in primary cortical neurons (Huang et al., 2018). We thus selected the optimal doses of NBP and NAD^+^ for ischemic stroke in mice and in primary neurons (Figures 3A, F). NBP 30 mg/kg and NAD^+^ 50 mg/kg was treated as soon as possible after reperfusion and the neuroprotective effects of the two drugs were compared. TTC staining results indicated that compared with the vehicle, both NBP and NAD^+^ treatment decreased the cerebral infarct volume of mice. Cerebral infarct volume was similarly reduced with NBP compared with NAD^+^ administration (Figures 3B, C). Compared with the vehicle, both NBP and NAD^+^ administration significantly improved the neurological scores (Figure 3D) and reduced the cerebral water content (Figure 3E) in mice with t-MCAO/R. The primary cortical neurons were treated with NBP and NAD^+^ after OGD/R immediately. Both NAD^+^ and NBP can increase the cell viability of neurons (Figure 3G).

### 3.3 Effects of NAD^+^ and NBP on long-term survival and neurological recovery after cerebral I/R in mice

**FIGURE 4:**
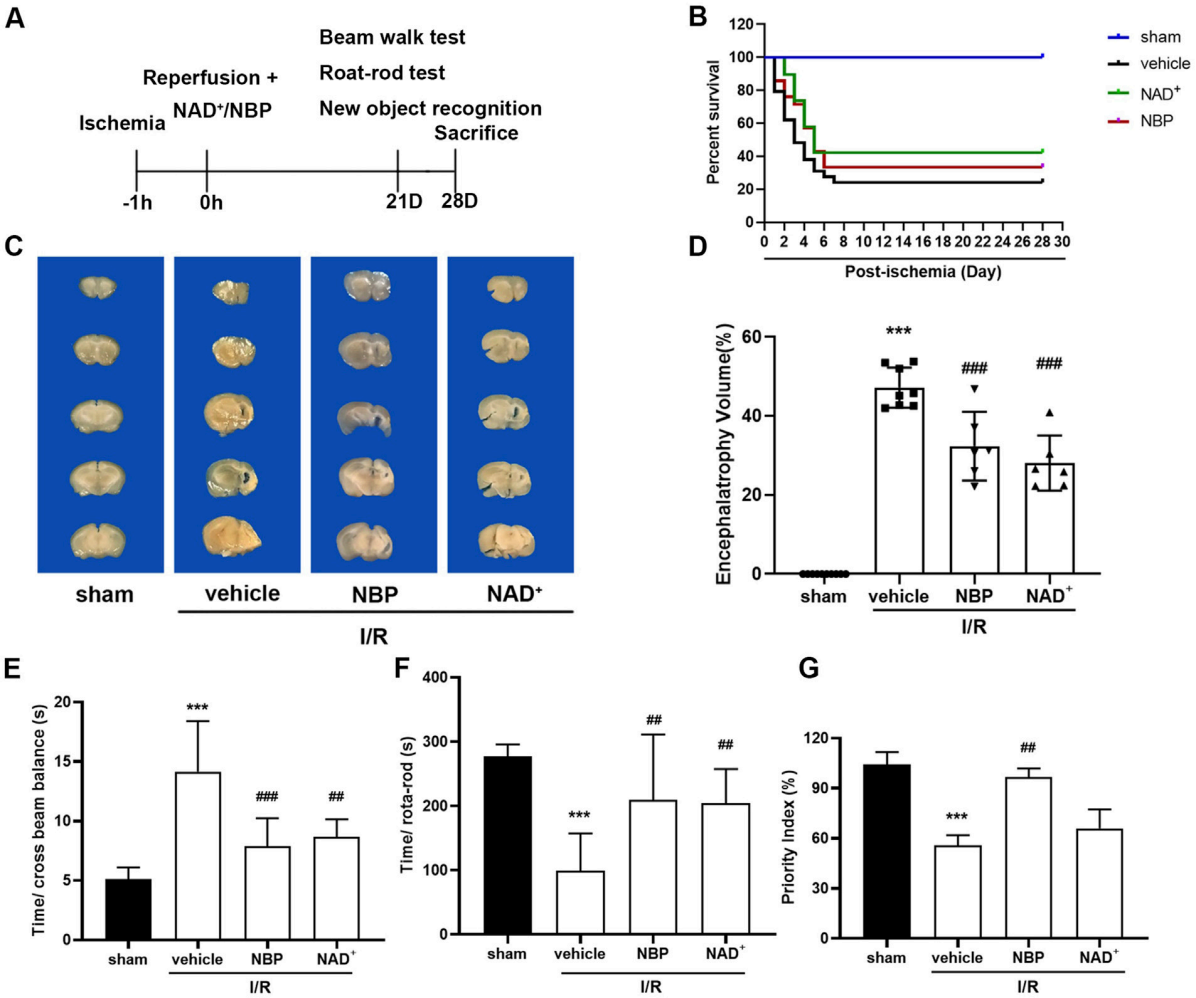
Effects of NAD^+^ and NBP on long-term survival and neurological recovery after cerebral I/R in mice. **(A)** Mice were treated to behavioral tests at the third week after I/R, and brain samples were collected at the fourth week for pathological analysis. **(B)** Ratio of mice surviving at 28 days after I/R surgery. **(C, D)** After treatment with NAD^+^ and NBP, brain atrophy of the ischemic hemisphere is decreased. NAD^+^ and NBP reduced the brain atrophy of the ischemic hemisphere 28 days after I/R. The effects of NAD^+^ and NBP on beam balance test **(E)**, rota-rod test **(F)** and new object recognition experiment **(G)** were detected at day 21 after I/R. ***p < 0.001 vs. sham, ^*##*^ *p <* 0.01 and ^*###*^ *p <* 0.001 vs. vehicle by One-way analysis of ANOVA followed by Tukey’s multiple comparisons test. Data were expressed as the mean ± SD. There were at least six animals in each group.

To further investigate the effects between NAD^+^ and NBP on long-term survival and neurological recovery after cerebral I/R in mice, we tested the survival, balance motor ability, coordination ability and learning and memory of the ischemic stroke mice within 28 days after surgery (Figure 4A). The results demonstrated that the survival of mice was only 24% after I/R 28 days, while that was 33% in NBP group and 42% in NAD^+^ group, respectively (Figure 4B). Compared to sham group, the brain atrophy in ischemic hemisphere of the model mice was robust, reaching 48% (Figures 4C, D), and both NAD^+^ and NBP could alleviate the brain atrophy. We performed behavioural tests on the mice with cerebral ischemia. The tests of balance beam (Figure 4E) and rota-rod rotation (Figure 4F) reflect the balance and coordination ability of mice. The results showed that both agents could restore the balance and coordination ability of ischemic mice. The learning and memory abilities of mice were also tested through new object recognition experiment (Figure 4G), and the results turned out NBP was better than NAD^+^ in improving the learning and memory ability of ischemic mice.

NBP and NAD^+^ showed similar antioxidant effects in cerebral I/R injury both *in vivo* and *in vitro*.

**FIGURE 5:**
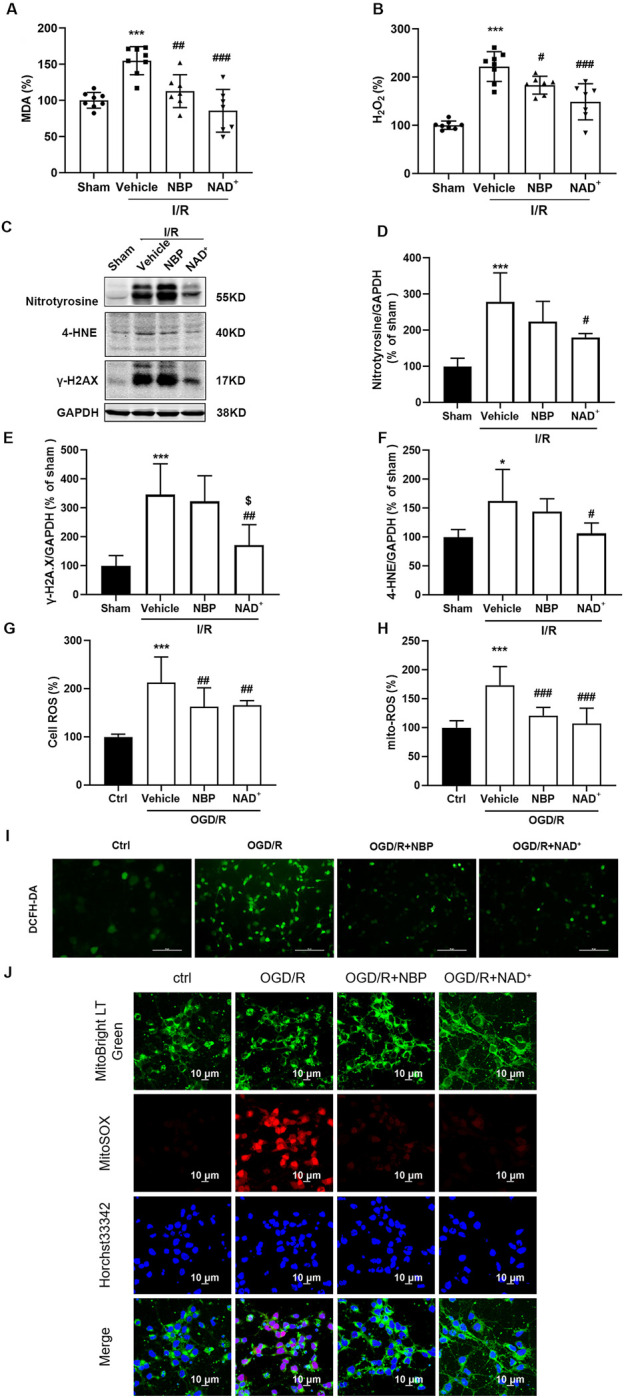
NBP and NAD^+^ showed similar antioxidant effects in cerebral I/R injury both *in vivo* and *in vitro*. **(A, B)** The effects of NAD^+^ or NBP on MDA **(A)**, H_2_O_2_ **(B)**. In mice, MCAO was performed for 2 h. NAD^+^ and NBP were treated immediately after reperfusion. The brain was removed at 24 h after reperfusion. **(C, D)** Analysis of nitrotyrosine expression in ischemic cortex. **(E)** Analysis of γ-H2A.X expression in ischemic cortex. **(F)** Analysis of 4-HNE expression in ischemic cortex. There were at least six animals in each group. **(G–J)** The primary cortical neurons were treated with NAD^+^ or NBP after OGD/R immediately. The ROS level in primary cortical neurons was detected with DCFH-DA green fluorescent probe **(G, I)** or MitoSOX red fluorescent probe **(H, J)**. The mitochondria were indicated by green LT fluorescent probe and the nucleus was indicated by Hoechst 33342. n = 10 per group, scale bar = 10 μm *p < 0.05, ***p < 0.001 vs. ctrl, ^*#*^ *p <* 0.05, ^*##*^ *p <* 0.01 and ^*###*^ *p <* 0.001 vs. vehicle, ^*$*^ *p <* 0.05 vs. NBP by One-way analysis of ANOVA followed by Tukey’s multiple comparisons test. Data were expressed as the mean ± SD.

Cerebral ischemia/reperfusion may cause oxidative stress, leading to a large amount of ROS production (Granger and Kvietys, 2015; Zhang et al., 2019). An important indicator of the intensity and rate of lipid peroxidation is malondialdehyde (MDA). We measured the contents of MDA and H_2_O_2_ in the cerebral cortex of mice after cerebral reperfusion. Compared with the sham group, the contents of MDA (Figure 5A) and H_2_O_2_ (Figure 5B) in the cerebral cortex of mice after t-MCAO/R were significantly increased, while both NAD^+^ and NBP could significantly reduce the contents of MDA and H_2_O_2_. Lipid peroxidation, protein nitration, and DNA damage can be induced by oxidative stress, leading to cell death (Nash, Schiefer, and Shah, 2018). In comparison with the sham group, the level of nitro-tyrosine (nitrotyrosine), a marker of protein nitration mediated by nitrogen monoxide (NO), was significantly increased 24 h after reperfusion. Similarly, the DNA damage marker protein γ-H2A.X (Zhang et al., 2019) and lipid peroxidation product 4-hydroxynonenaldehyde (4-HNE) (Guo et al., 2013) increased 24 h after reperfusion. However, compared to vehicle group, the protein levels of nitrotyrosine (Figures 5C, D), γ-H2A.X (Figure 5E) and 4-HNE (Figure 5F) were significantly downregulated with NAD^+^ treatment, while NBP treatment had no significant effect on these parameters. Using DCFH-DA (Figures 5G, I) and mitochondrial superoxide fluorescence staining (Figures 5H, J) in primary cortical neurons subjected to OGD/R, both NAD^+^ and NBP could significantly inhibit the accumulation of ROS and mitochondrial superoxide. The above data indicated that NAD^+^ showed slightly better antioxidant effect than that of NBP in cerebral I/R injury.

### 3.4 NAD^+^ is superior to NBP in improving energy imbalance after ischemia/reperfusion

**FIGURE 6:**
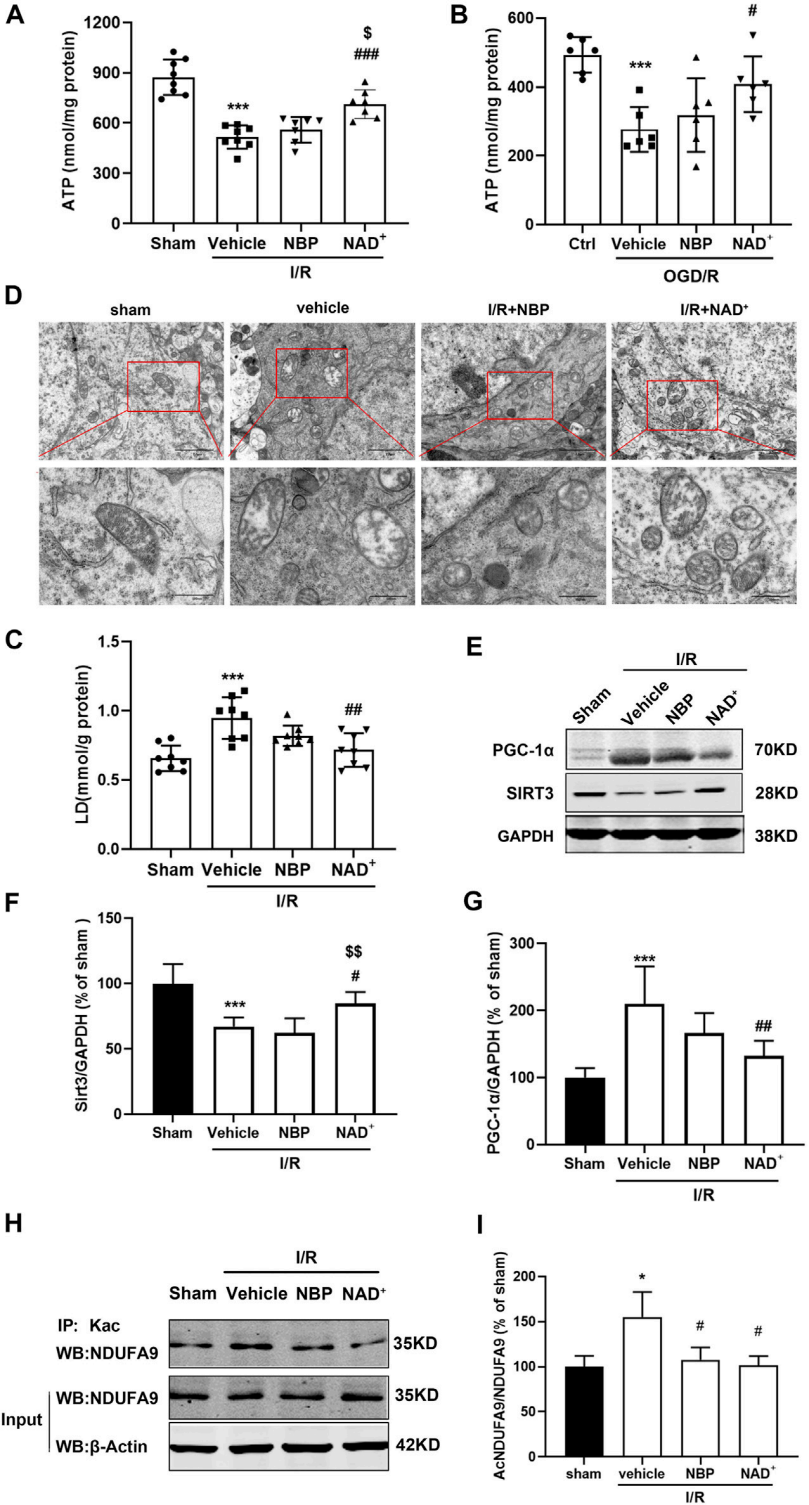
NAD^+^ is superior to NBP in improving energy metabolism. **(A)** In mice, MCAO was performed for 2 h. NAD^+^ and NBP were administered immediately after reperfusion. ATP levels in cortex 3 h after cerebral ischemia/reperfusion in mice. **(B)** The primary cortical neurons were treated with 5 μM NBP and 15 mM NAD^+^ after OGD/R immediately. Neuronal ATP levels were detected with ATP detection kits 3 h after OGD/R. **(C)** LD levels in cortex 3 h after cerebral ischemia/reperfusion in mice. **(D)** Mitochondrial morphology in ischemic region was observed by transmission electron microscopy after I/R. Scale bar = 500 nm. The brain was removed at 24 h after reperfusion. **(E, F)** Analysis of SIRT3 expression in ischemic cortex. **(G)** Analysis of PGC-1α expression in ischemic cortex. **(H, I)** Analysis of acetylation of NDUFA9 in ischemic cortex. ***p < 0.001 vs. sham or ctrl, ^*#*^ *p <* 0.05, ^*#*^ *p <* 0.05, ^*##*^ *p <* 0.01 and ^*###*^ *p <* 0.001 vs. vehicle, ^*$*^ *p <* 0.05, ^*$$*^ *p <* 0.01vs. NBP by One-way analysis of ANOVA followed by Tukey’s multiple comparisons test. Data were expressed as the mean ± SD. There were at least six animals or three independent cultures in each group.

The depletion of NAD^+^ or the imbalance of NAD^+^/NADH can cause mitochondrial dysfunction, leading to blockade of ATP synthesis (Cantó et al., 2015). We measured the ATP levels and lactic acid (LD) contents in the injured cortex of mice after cerebral I/R. The results indicated that the levels of ATP decreased (Figure 6A) and the levels of LD increased (Figure 6C) in the cerebral cortex after reperfusion. A decrease in ATP and increase in LD may be reversed through NAD^+^, while NBP had no significant effect on these changes (Figure 6A). Similar improvement on ATP recovery was found in the cortical neurons treated with NAD^+^ instead of NBP after OGD/R *in vitro* (Figure 6 B). We observed the mitochondrial morphology in neurons in the cerebral cortex of mice with ischemic stroke through transmission electron microscopy. The results showed that cerebral ischemia-reperfusion caused severe mitochondrial damage, as evidenced by obvious mitochondrial swelling and vacuolation, while treatment with NAD^+^ and NBP could significantly improve mitochondrial morphology (Figure 6D).

SIRT3 is an important NAD^+^ - dependent deacetylase in mitochondria, which can regulate the activities of many metabolic enzymes in mitochondria through deacetylation (Nash et al., 2018; Zhang et al., 2019). In SIRT3-deficient mice, mitochondrial respiratory chain complex subunit 9 (NDUFA9) was excessively acetylated, resulting in decreased mitochondrial oxygen consumption and ATP synthesis. Peroxisome proliferator-activated receptor-γ coactivator -1α (PGC-1α), a mitochondrial biogenesis related protein, acts as an inducible co-regulator in regulating energy homeostasis and is highly expressed in tissues with high energy requirements (Yu et al., 2017). We measured the effects of NAD^+^ or NBP on these mitochondrial protein expression in the cortex of the mice with ischemic stroke. Compared to vehicle group, NAD^+^ could increase the protein levels of SIRT3, while NBP had no effect on SIRT3 (Figures 6D, E). Similarly, NAD^+^ reduced the levels of PGC-1α, but NBP had no significant effect on PGC-1 α level (Figure 6F). However, both NAD^+^ and NBP significantly decreased the acetylation of NDUFA9 (Figure 6G). These results suggest that NAD^+^ may deacetylated NDUFA9 by increasing SIRT3 activity, thus improving mitochondrial function and antioxidant effects. However, NBP protects cerebral ischemia-reperfusion injury mainly not through SIRT3 to improve mitochondrial function.

Effects of sirtuin inhibition on the protection of NAD^+^ and NBP in cerebral ischemia.

**FIGURE 7:**
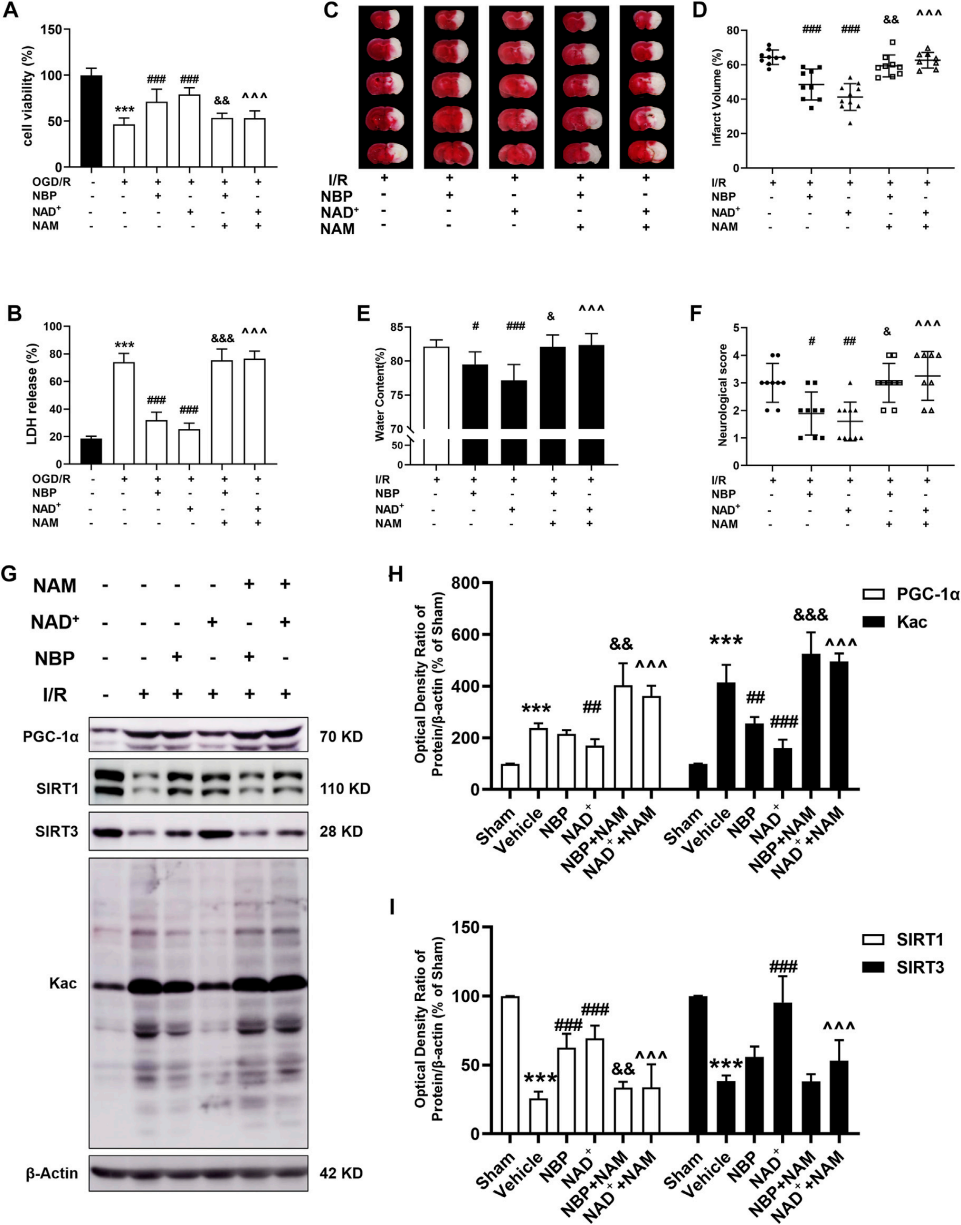
Effects of sirtuin inhibitor on the protection of NAD^+^ and NBP in cerebral ischemia. **(A, B)** Primary cortical neurons were treated with 10 mM NAM for 4 h. Glucose and hypoxia were next taken away from the primary cortical neurons for 4 h, and then 15 mM NAD^+^ or 5 μM NBP were administered transiently after reperfusion. After 24 h of reperfusion, the viability and toxicity of the cells was determined using the CCK-8 kit and LDH assay kit. **(A)** Cell viability in each group. **(B)** Cell toxicity in each group. n = 6 per group. **(C–F)** In mice, NAM (100 mg/kg) were administrated 2 h before tMCAO. tMCAO was next performed for 2 h, and then NBP (30 mg/kg) or NAD^+^ (50 mg/kg) were administrated to mice immediately after reperfusion. tMCAO/R-treated mice were sacrificed 24 h after the procedure. **(C, D)** TTC staining was used to measure the cerebral infarct volume. **(E)** The brain water content at 24 h after reperfusion. **(F)** The neurological deficit score at 24 h after reperfusion. **(G–H)** Analysis of PGC-1α and Kac expression in ischemic cortex. **(I)** Analysis of SIRT1 and SIRT3 expression in ischemic cortex. ***p < 0.001 vs. sham, ^*#*^ *p <* 0.05, ^*#*^ *p <* 0.05, ^*##*^ *p <* 0.01 and ^*###*^ *p <* 0.001 vs. vehicle + I/R or vehicle + OGD/R, ^*&*^ *p <* 0.05, ^*&&*^ *p <* 0.01 and ^*&&&*^ *p <* 0.001vs. NBP, ^*^^^*^ *p <* 0.001vs. NAD^+^ by One-way analysis of ANOVA followed by Tukey’s multiple comparisons test. Data were expressed as the mean ± SD. There were at least six animals or three independent cultures in each group.

To determine whether NAD^+^ or NBP can improve cerebral ischemia by regulating sirtuin, we used the sirtuin inhibitor NAM to observe changes in the protective effects of NAD^+^ or NBP. Primary cortical neurons were treated with 10 mM NAM for 4 h, after which OGD/R was performed, and either NAD^+^ or NBP was administered after reperfusion. The results showed that NAD^+^ or NBP had a protective effect on neuronal OGD/R injury, increasing cell viability and decreasing LDH release, but NAM pretreatment abolished the protection of NAD^+^ or NBP on neuronal OGD/R injury (Figures 7A, B). Mice were pretreated with NAM (100 mg/kg) 2 h before tMCAO/R, and NBP (30 mg/kg) or NAD^+^ (50 mg/kg) were administrated immediately after reperfusion. TTC staining indicated that NAM treatment before either NBP or NAD^+^ administration increased the cerebral infarct volume in I/R mice (Figures 7C, D). NAM treatment before NBP or NAD^+^ administration significantly increased the cerebral water content (Figure 7E) and the neurological scores (Figure 7F) in mice compared with the NBP group. To determine whether NAD^+^ or NBP could ameliorate mitochondrial dysfunction and protect against cerebral ischemia by modulating sirtuin, we determined the effect of NAM on cortical protein expression in mice with ischemic stroke after NAD^+^ or NBP treatment (Figure 7G). Consistent with the above data, NAD^+^ but not NBP significantly decreased PGC-1α and increased SIRT3 expression, whereas NAM treatment significantly increased PGC-1α and decreased SIRT3 expression compared with the NAD^+^ group (Figures 7H, I). Interestingly, both NAD^+^ and NBP ameliorated I/R-induced SIRT1 reduction, while NAM pretreatment reduced SIRT1 levels compared with NAD^+^ or NBP treatment groups. Consistently, NAD^+^ or NBP significantly decreased I/R-induced pan-acetylation of cortical proteins, whereas NAM increased protein acetylation compared with NAD^+^ or NBP groups (Figures 7H, I). These results suggest that NAD^+^ may reduce protein acetylation *via* SIRT1 and SIRT3, while NBP may reduce acetylation *via* SIRT1, to ameliorate mitochondrial dysfunction and oxidative stress in cerebral ischemic injury. Sirtuin inhibitor NAM intervention thus can abrogate the protective effect of NAD^+^ or NBP against cerebral ischemic injury.

## 4 Discussion

Stroke is the second major cause of death and disability in the world (Klimova et al., 2019), so the development of effective therapeutic drugs is extremely urgent. NAD^+^ is an important coenzyme to mediate H^+^ transport in many metabolic reactions in cells. In the glycolysis pathway, the oxidative decomposition of glucose to pyruvate is accompanied by the conversion of NAD^+^ to NADH (Cai et al., 2021). In the mitochondrial tricarboxylic acid cycle, acetyl CoA is oxidized to carbon dioxide, accompanied by the conversion of NAD^+^ to NADH. The transformation of NAD^+^ to NADH also exists in the oxidation of mitochondrial fatty acids and amino acids. NADH is the electron donor for mitochondrial oxidative phosphorylation and ATP synthesis (Imai and Guarente, 2014). It has been reported that supplementation with exogenous NAD^+^ or its precursors can improve mitochondrial function (Cai et al., 2021) and prevent the occurrence and development of some neurodegenerative diseases (Xu et al., 2012). Our previous study demonstrated that exogenous NAD^+^ administration can improve cerebral ischemia-reperfusion injury in mice. In addition, it has been reported that NBP can ameliorate cerebral ischemia/reperfusion injury in rats by facilitating vascular regeneration (Li et al., 2010; Yang et al., 2015) and neural restoration (Yin et al., 2017; Peng et al., 2020). Consistent with these studies, our current results confirmed that NBP can effectively reduce the neuronal injury caused by cerebral ischemia/reperfusion both *in vivo* and *in vitro*.

In this study, ICR mice were subjected to t-MCAO/R, followed by exogenous NAD^+^ or NBP immediately after reperfusion. Through TTC staining, we found that both NAD^+^ and NBP significantly reduced infarct volume, cerebral edema, and neurological deficits in mice. In addition, in the primary cortical neurons, NAD^+^ and NBP could also protect the neurons against OGD/R injury, which was consistent with the study *in vivo*.

Clinically, the thrombolytic drugs tend to miss the optimal time in the treatment of ischemic stroke (Paul and Candelario-Jalil, 2021), so the therapeutic time window for cerebral ischemia is also an important indicator to evaluate the efficacy of drugs. In the study, we investigated the time window of NBP against cerebral ischemia, and found that compared with NAD^+^, NBP had a longer therapeutic time window and might be more advantageous in the treatment of stroke patients.

Long-term survival and neurological recovery in stroke patients are the important indicators of clinical treatment. Therefore, we compared those effects of NAD^+^ and NBP on mice. We found that both NAD^+^ and NBP improved the 28-day survival rate of mice subjected to I/R and reduced the brain atrophy volume of the mice. In terms of neurological recovery, there was no significant difference between NAD^+^ and NBP in restoring the balance and coordination ability of mice, but NBP seems to be superior to NAD^+^ in restoring the learning and memory ability of mice.

During cerebral ischemia/reperfusion, glucose, and oxygen deficiency may induce mitochondrial dysfunction, leading to oxidative stress and reduced ATP synthesis (Ahn et al., 2008; Salvatori et al., 2017; Liu et al., 2019). In this study, we compared the effects of NAD^+^ and NBP on oxidative stress and energy metabolic homeostasis. The data showed that both NAD^+^ and NBP could reduce the content of MDA and H_2_O_2_ after cerebral ischemia/reperfusion. Our study also found that with the time of cerebral ischemia reperfusion in mice, the protein levels of nitrotyrosine (marker of protein nitrification mediated by nitric oxide), 4-HNE (marker of lipid peroxidation), and γ-H2A.X (marker of DNA damage), gradually increased with the most significant at 24 h after reperfusion (Data not shown). Interestingly, NAD^+^ treatment significantly reduced 4-HNE and γ-H2A.X levels after cerebral ischemia/reperfusion, while NBP had no significant effect on these indexes. Consistent with *in vivo* results, DCFH-DA and mitochondrial ROS staining of primary cortical neurons after OGD/R *in vitro* showed that both NAD^+^ and NBP had antioxidant ability. These results suggested that antioxidant activity of NAD^+^ was a slightly better than that of NBP in rodent model of ischemic stroke.

After cerebral ischemia and reperfusion, the intracellular NAD^+^/NADH pool is out of balance, and the NAD^+^ level is decreased. NAD^+^ is an important cofactor in cellular metabolism, and the deacetylation of SIRT3 depends on NAD^+^(Salvatori et al., 2017), the activity of SIRT3 is inhibited (Hathorn et al., 2011), which in turn inhibits the deacetylation of its downstream proteins such as SOD2, PGC-1α, AMPK, leading to increased acetylation of these proteins. This may lead to mitochondrial dysfunction, increase mitochondrial ROS production of and decrease ATP production. We thus further investigated whether cerebral ischemia reperfusion regulates SIRT3 to cause mitochondrial dysfunction and energy metabolism dysfunction. NAD^+^ significantly reversed the decrease of ATP and increase of LD after cerebral ischemia reperfusion, but NBP had no significant effect on the production of ATP and LD. These results suggest that NAD^+^ might be superior to NBP in improving energy imbalance caused by ischemia-reperfusion. However, transmission electron microscopy observation of the mitochondrial morphology in the ischemic cortex of the ischemic stroke mice showed that both NAD^+^ and NBP reduced mitochondrial vacuolation/swelling and mitochondrial crest rupture induced by ischemia-reperfusion. We further analysed the mitochondrial related proteins in the cerebral cortex of mice, and found that with the increase of reperfusion time, the level of SIRT3 was gradually downregulated, while the expression of PGC-1α was continuously increased. After reperfusion for 24 h, the expression of SIRT3 and PGC-1α changed most significantly (Data not shown). NAD^+^ treatment inhibited the changes of SIRT3 and PGC-1α, but NBP did not significantly improve their changes. In addition, after cerebral ischemia reperfusion, acetylation of mitochondrial respiratory chain complex subunit 1 NDUFA9 was significantly upregulated, while NAD^+^ and NBP significantly decreased the acetylation of NDUFA9, suggesting that both NAD^+^ and NBP could facilitate the mitochondrial respiratory chain to improve mitochondrial function. Literature shows that SIRT3 can activate NDUFA9 through deacetylation to regulate mitochondrial respiration and to improve mitochondrial function (Lang et al., 2015). NBP protects neuronal death by restoring cerebral blood flow and microcirculation in the ischemic area, alleviating mitochondrial dysfunction, and reducing neuroinflammation (Yang et al., 2019). We thus assume that NAD^+^ might improve mitochondrial function by increasing the activity of SIRT3 and then deacetylating NDUFA9, but the mechanism by which NBP improves mitochondrial function and deacetylates NDUFA9 may be independent of SIRT3.

We further used the sirtuin inhibitor NAM to verify the relationship between the neuroprotection of NAD^+^ or NBP and SIRT1 or SIRT3. The results showed that intervention with NAM prior to NAD^+^ and NBP treatment reduced neuronal viability, increased LDH release from neurons after OGD/R. NAM also increased cerebral infarct volume in mice, brain water content and neurological deficit of t-MCAO/R mice, suggesting that NAM could eliminate the protective effect of NAD^+^ or NBP on cerebral ischemia. NAM treatment before NAD^+^ and NBP administration significantly increased NAD^+^ and NBP-induced PGC-1α reduction and pan-acetylation of cortical proteins. Under ischemia/hypoxia, the decreased deacetylation activity of SIRT1 causes the acetylation of downstream targets, leading to a series of cellular damage processes (Meng et al., 2017). Decreased SIRT1 activation activates PGC-1α and uncoupling protein-2 (UCP2), which promotes the generation of reactive oxygen species. SIRT1 reduction also activates the NF-κB pathway, leading to inflammatory responses (Wang et al., 2009). Furthermore, SIRT1 reduction also causes acetylation of forkhead box O (Foxos) or p53, leading to apoptosis (Dioum et al., 2009). Interestingly, low levels of NF-κB were associated with upregulation of AMPK/SIRT1 after NBP treatment, suggesting that NBP may function as a regulator between inflammation and AMPK/SIRT1 pathway (Li et al., 2020). In addition, NBP reversed the decrease of SIRT1 and PGC-1α and inhibited cell apoptosis in Chronic intermittent hypoxia hypercapnia (CIHH). NBP thus exerts neuroprotective effect by activating SIRT1/PGC-1α pathway (Min et al., 2014). Our study showed that although NAD^+^ but not NBP upregulated SIRT3 protein expression, both NAD^+^ and NBP upregulated SIRT1 and reduced panacetylation of cortical proteins. NAM intervention cancelled the upregulation of SIRT1 and SIRT3 and increased cortical protein panacetylation. These results suggest that NBP may act through SIRT1, while NAD^+^ may act through SIRT1 and SIRT3 to restore mitochondrial respiratory function, improve energy metabolism and inhibit oxidative stress during cerebral ischemia reperfusion. Further research on the neuroprotective mechanisms of NAD^+^ and NBP is still necessary in the future.

In conclusion, the results of this study demonstrated that NAD^+^ and NBP showed similar neuroprotective effects on cerebral ischemia-reperfusion injury, but the therapeutic window of NBP was longer than that of NAD^+^. Furthermore, NBP and NAD^+^ showed similar antioxidant effects, while NAD^+^ is superior to NBP in restoring the balance of energy metabolism, possibly through regulating the activity of SIRT1 and SIRT3. The protective effect of NBP on cerebral ischemia/reperfusion may be achieved through SIRT1.

## Data Availability

The original contribution presented in the study are included in the article/supplementary material, further inquiries can be directed to the corresponding authors.
